# Sustainability in Analytical Chemistry Illustrated by Pharmaceutical Nitrosamine Testing

**DOI:** 10.1002/elps.70067

**Published:** 2025-12-24

**Authors:** Felix Bredendiek, Sebastian Schmidtsdorff, Maria Kristina Parr

**Affiliations:** ^1^ Department of Biology, Chemistry, Pharmacy, Institute of Pharmacy Freie Universität Berlin Berlin Germany; ^2^ Department of Biology, Chemistry, Pharmacy, Core Facility BioSupraMol Freie Universität Berlin Berlin Germany; ^3^ Department Risk Communication German Federal Institute for Risk Assessment GLP Federal Bureau Berlin Germany

**Keywords:** gas chromatography (GC), green chemistry, HPLC, MS, *N*‐nitrosamines, supercritical fluid chromatography (SFC), sustainability

## Abstract

Following the valsartan scandal in 2018, the testing of drug substances and drug products for *N*‐nitrosamines has become a critical and mandatory quality control measure. The European Pharmacopoeia chapter 2.5.42 currently describes three analytical methods for this purpose: HPLC–MS/MS, GC–MS, and GC–MS/MS. The US Pharmacopeia monograph 〈1469〉 adds four other methods, LC–high‐resolution mass spectrometry (HRMS), headspace GC–MS, LC–MS/MS, and GC–MS/MS. In addition, our group has developed a universal method on the basis of supercritical fluid chromatography (SFC), capable of separating 16 different *N*‐nitrosamines within just 4 min. These eight methods differ significantly in terms of sustainability, with particular emphasis on the reagents used, the separation techniques employed, and their performance characteristics. When assessing the sustainability of such analytical methods, it is essential to consider not only ecological but also economic factors.

**Abbreviations**
AGREE: Analytical GREEnness Metrics
CLND: chemiluminescence nitrogen detection
CRS: chemical reference substances
HRMS: high‐resolution mass spectrometry
HS‐GC: headspace gas chromatography
NDBA: *N*‐nitrosodibutylamine
NDEA: *N*‐nitrosodiethylamine
NDiPA: *N*‐nitrosodiisopropylamine
NDMA: *N*‐nitrosodimethylamine
NDPA: *N*‐nitrosodipropylamine
NEiPA: *N*‐nitrosoethylisopropylamine
NEMA: *N*‐nitrosoethylmethylamine
NMBA: *N*‐nitroso‐*N*‐methyl‐4‐aminobutyric acid
NMPA: *N*‐nitroso‐*N*‐methylphenylamine
NPD: nitrogen‐phosphorous detection
Ph. Eur.: European Pharmacopoeia
QC: quality control
SDG: Sustainable Development Goal
SFC: supercritical fluid chromatography
SST: system suitability test
TEA: thermal energy analyzer
USP: US Pharmacopoeia

## 1 Introduction

**FIGURE 1:**
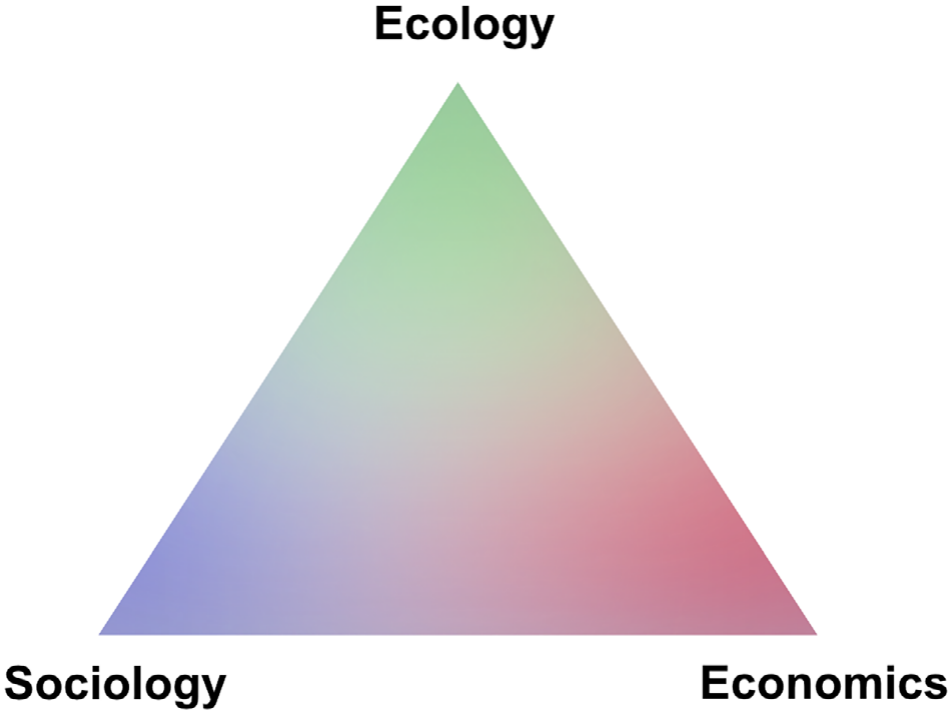
Sustainability illustrated as triangle of the competing priorities: ecology, economics, and sociology.

The world is changing, and one of the most important issues that needs to be addressed is sustainability. The “One Health” concept tries to address this by an integrative approach that sees human, animal, and environmental health as inextricably linked. For this purpose, the United Nations Sustainable Development Goals (SDGs) provide an important framework for achieving these global health, environment, and development goals together [1, 2, 3]. This applies not only to our daily lives but also specifically to the pharmaceutical industry and analytical chemistry. Thus, these concepts can be implemented into pharmaceutical analysis, for example, by incorporating sustainability as a key factor during method development. On the one hand, there are obvious questions, such as: How can the ecological impact be minimized? On the other hand, it is equally important not to overlook the influence that ecological considerations can have on economic factors and the analytical efficiency of a method. These connections are illustrated in Figure 1.

Over the years, the term “green chemistry” has become a broad concept for optimizing chemical processes in the context of environmental sustainability [4]. This includes replacing harmful chemicals, negatively impacting environmental and human health, as well as optimizing energy consumption.

In chemical and pharmaceutical analytics, the term “green analytical chemistry” evolved thereof [5, 6, 7]. Here, 12 criteria are considered, adapted from the principles of green chemistry. At present, various evaluation systems are used to better assess these different criteria [8]. One of the most commonly used systems appears to be the Analytical GREEnness Metric (AGREE) system. In this system, each criterion is rated with a score between 0 (not sustainable) and 1 (excellent sustainability) [9]. Additionally, the criteria can be weighted according to their relevance. From all the scores, including the weightings, a final overall score is calculated. In addition to numerical scoring, a color code (red–yellow–green) is used to provide a quick and easy visual assessment.

In the following, the evaluation of method greenness is illustrated using the AGREE system. For illustration, a comparison of methods for nitrosamine analysis with a focus on green analytical chemistry was conducted. Specifically, we aim to compare the methods described in the European Pharmacopoeia (Ph. Eur.) [10] and US Pharmacopoeia (USP) [11] with a newly developed supercritical fluid chromatography (SFC) method by Schmidtsdorff et al. [12].

After the nitrosamine crisis in 2018, the importance of testing for these substances became increasingly clear [13]. Nitrosamines are a class of chemical compounds that have raised significant public health concerns due to their potential carcinogenicity. Nitrosamines are commonly found in tobacco smoke, certain foods, and industrial processes but can also be produced unintentionally during drug manufacturing. Their importance in the pharmaceutical industry came to global attention during the valsartan scandal in 2018, when several batches of the widely used antihypertensive drug valsartan were found to be contaminated with nitrosamines such as *N*‐nitrosodimethylamine (NDMA). This discovery led to widespread recalls, increased regulatory scrutiny, and a re‐evaluation of manufacturing practices, underscoring the critical need for strict quality control (QC) in drug manufacturing to protect public health [14, 15]. Since 2019, testing for nitrosamines in drug substances and drug products became mandatory on the basis of international regulatory guidelines [16, 17, 18]. With the introduction of section 2.5.42 (*N*‐Nitrosamines in active substances) [10] and the USP chapter 〈1469〉 (Nitrosamine impurities) [11] in 2021, the previously individual respective tests for *N*‐nitrosamines in the monographs were replaced by a general reference to these sections.

The Ph. Eur. describes three specific methods for testing drug substances for *N*‐nitrosamines, utilizing HPLC–MS/MS, GC–MS, or GC–MS/MS. The three methods together test for the following seven *N*‐nitrosamines in sartan drug substances: NDMA, *N*‐nitrosodiethylamine (NDEA), *N*‐nitrosodibutylamine (NDBA), *N*‐nitroso‐*N*‐methyl‐4‐aminobutyric acid (NMBA), *N*‐nitrosodiisopropylamine (NDiPA), *N*‐nitrosoethylisopropylamine (NEiPA), and *N*‐nitrosodipropylamine (NDPA). The specific number of impurities tested varies depending on the specific method. In contrast, the USP describes four methods, also based on separation using GC or HPLC coupled with mass spectrometric detection. Differences are found in the injection technique (headspace injection) and the type of mass spectrometry used (high‐resolution mass spectrometry [HRMS]). A total of seven impurities are tested using the USP methods: NDMA, NDEA, NEiPA, NDiPA, NDBA, NMBA, and *N*‐nitroso‐*N*‐methylphenylamine (NMPA). A comparison of the impurities tested in the different methods from Ph. Eur. and USP is shown in Table 1.

**TABLE 1:** Application of the methods from Ph. Eur. (A, B, C) and USP (P1‐4), * in Method A, the presence of DMF (dimethylformamide) in the substance to be tested may interfere with the detection of NDMA.

Drug substance	*NDMA*	*NDEA*	*NDBA*	*NMBA*	*NDiPA*	*NEiPA*	*NDPA*	*NMPA*
Candesartan cilexetil	A*, B, C P2, 3, 4 SFC	A, B, C P2, 3, 4 SFC	C P3, 4 SFC	A P3 SFC	A, C P2, 3, 4 SFC	A, C P2, 3, 4 SFC	C SFC	P4 SFC
Irbesartan	A*, B, C P1, 2 SFC	A, B, C P1, 2 SFC	C P1 SFC	A P1 SFC	A, C P1, 2 SFC	A, C P1, 2 SFC	C SFC	P1 SFC
Losartan potassium	A*, B, C P1, 2, 3, 4 SFC	A, B, C P1, 2, 3, 4 SFC	C P1, 3, 4 SFC	A P1, 3 SFC	A, C P1, 2, 3, 4 SFC	A, C P1, 2, 3, 4 SFC	C SFC	P1, 4 SFC
Olmesartan medoxomil	A*, B, C P2, 3 SFC	A, B, C P2, 3 SFC	C P3 SFC	A P3 SFC	A, C P2, 3 SFC	A, C P2, 3 SFC	C SFC	SFC
Valsartan	A*, B, C P1, 2, 3, 4 SFC	A, B, C P1, 2, 3, 4 SFC	C P1, 3, 4 SFC	A P1, 3 SFC	A, C P1, 2, 3, 4 SFC	A, C P1, 2, 3, 4 SFC	C SFC	P1, 4 SFC
Telmisartan	P2, 3 SFC	P2, 3 SFC	P3 SFC	P3 SFC	P2, 3 SFC	P2, 3 SFC	SFC	SFC

*Note*: Note that the SFC method of Schmidtsdorff et al. [12] covers all the eight listed nitrosamines and eight more. Additionally, it is universally applicable to all kinds of drug substances and drug products that are not explicitly mentioned here.

Abbreviations: NDBA, *N*‐nitrosodibutylamine; NDEA, *N*‐nitrosodiethylamine; NDiPA, *N*‐nitrosodiisopropylamine; NDMA, *N*‐nitrosodimethylamine; NDMA, *N*‐nitrosodimethylamine; NDPA, *N*‐nitrosodipropylamine; NEiPA, *N*‐nitrosoethylisopropylamine; NMBA, *N*‐nitroso‐*N*‐methyl‐4‐aminobutyric acid; NMPA, *N*‐nitroso‐*N*‐methylphenylamine; Ph. Eur., European Pharmacopoeia; USP, US Pharmacopoeia.

*Source*: Table adapted from Ph. Eur. [10] and extended with data from USP [11].

In comparison to the methods in the pharmacopeial methods, Schmidtsdorff et al. used supercritical carbon dioxide as eluent in their separation method (SFC) with detection via MS/MS. Furthermore, the latter method detects a total of 16 *N*‐nitrosamines, based on the lists published by regulatory authorities with small aliphatic, cyclic, and aromatic nitrosamines [16, 18]. The method is universally applicable to both drug substances and drug products and can be easily extended to new impurities such as nitrosamine drug substance‐related impurities [19]. In the last years, many methods for the detection of *N*‐nitrosamines were reported, but only a few mentioned greenness or sustainability [20, 21]. Besides the already mentioned analytical methods using GC, HPLC, or SFC, capillary electrophoresis (CE) can also be a separation technique of choice for basic or acidic *N*‐nitrosamines. This was recently published for tobacco smoke [22] but may be transferable to drug analysis as well. In general, it is assumed that CE separation will be considered superior in terms of greenness. However, it needs to be considered that very low limits of quantitation will be needed, and thus, full method performance characterization will be needed to demonstrate its suitability for drug analysis.

## 2 Methodology

The investigation of sustainability measures in analytical chemistry is illustrated using selected methods for *N*‐nitrosamine testing in drug substances and drug products. An evaluation was carried out for eight different methods using different chromatographic separation techniques, each with mass spectrometric detection, meeting the currently applied requirements of regulatory authorities for Europe and United States. We also included a method from our research group in the comparison, which has been validated for a wide range of drug substances.

## 3 Results of Greenness Measures for the Compared Methods

**FIGURE 2:**
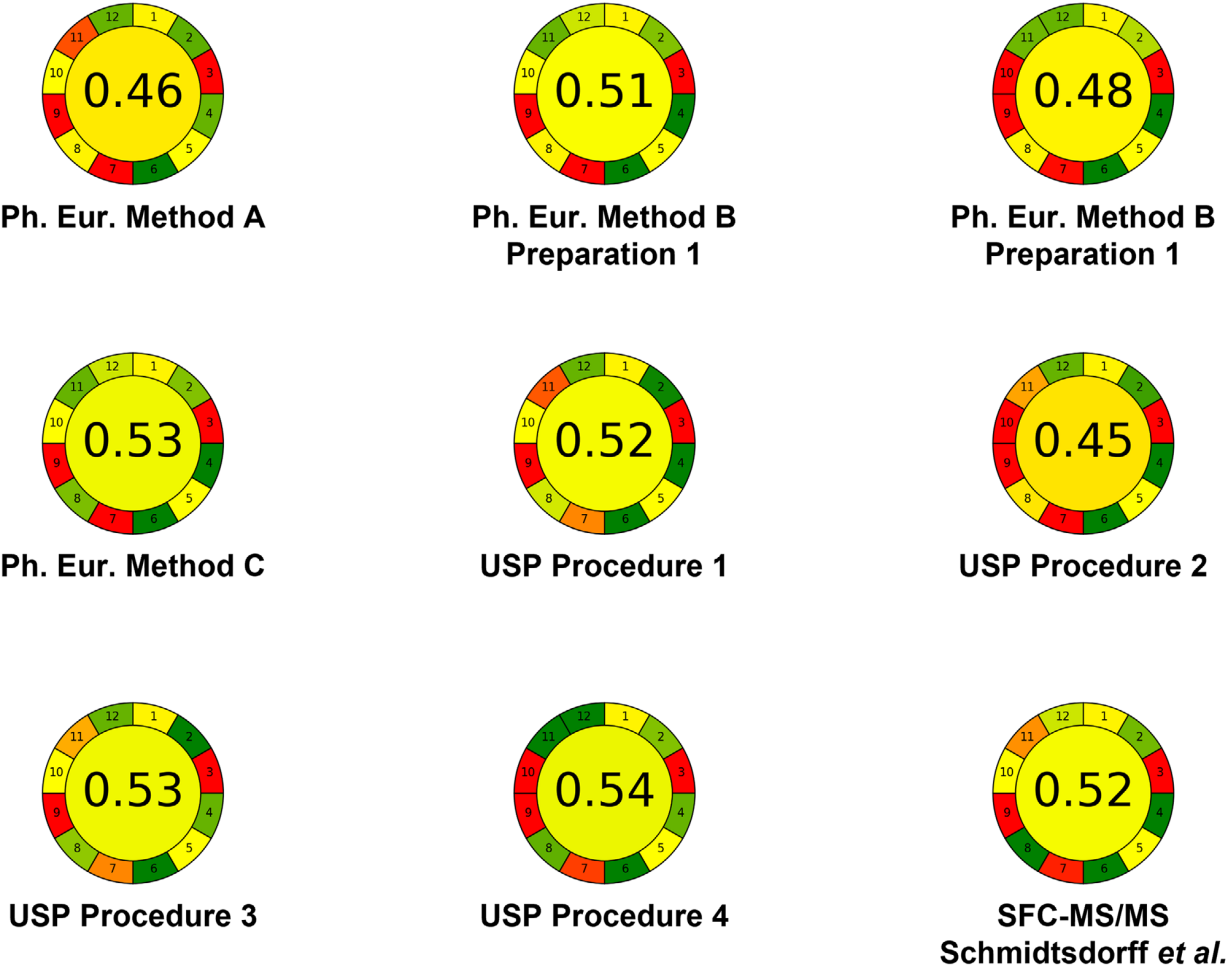
Results from AGREE characterization for Methods A, B (both sample preparations), and C from the Ph. Eur. 2.5.42; USP 〈1469〉 Procedures 1, 2, 3, and 4 as well as the SFC–MS/MS method by Schmidtsdorff et al. [1]. Ph. Eur., European Pharmacopoeia; SFC, supercritical fluid chromatography; USP, US Pharmacopoeia.

To facilitate a comparative analysis of the methods, the individual steps of each approach are considered in more detail. Figure 2 presents a comparison of the methods using the AGREE evaluation tool (application V 0.5). Individual input parameters are reported in the supplementary material. In addition to the eight distinct analytical methods considered, the GC–MS method described in the Ph. Eur. (Method B) includes two different sample preparation procedures: Preparation 1 (applicable to valsartan, losartan, and olmesartan) and Preparation 2 (applicable to candesartan and irbesartan). Although it would have been possible, we deliberately refrained from applying differential weighting to the 12 parameters. Consequently, in this comparison, each parameter contributes equally to the overall score. The overall sustainability score ranges from 0.45 (USP Procedure 2, GC–MS) to 0.54 (USP Procedure 4, GC–MS/MS).

In general, it should be noted that the four USP methods and the three Ph. Eur. methods are currently only applicable to sartan drug substances, and therefore, the calculated sustainability has its limitations, whereas the method of Schmidtsdorff et al. is universally suitable to various drug substances and drug products [23]. As no literature or press publications currently address this matter, the applicability of the USP or Ph. Eur. methods to other drug substances or drug products remains uncertain due to potential selectivity issues. However, it should be noted that such application is generally feasible, provided full method validation is performed and successful.

### 3.1 Sample Preparation

Method A of Ph. Eur., a semiquantitative LC–MS/MS limit test, employs a methanolic solution of NDEA‐d_9_ as internal standard, which is added to the test solution of the drug substance, the spiked drug substance test solution, and the reference solution. Additionally, for spiking the test solution and preparing the reference solution, a spike solution is prepared that contains all *N*‐nitrosamine impurities as chemical reference substances (CRS). The test solution, spiked test solution, and reference solution are prepared in the same manner. The spiked drug substance test solution is necessary due to the semiquantitative nature of limit tests. Here, only peak area ratios of the unspiked and the spiked drug substance test solution are evaluated to determine if the unspiked solution exceeds a certain threshold. Therefore, the threshold amount is spiked to a second test solution to retrieve the spiked test solution used for comparison with the non‐spiked. The reference solution is used as a system suitability test (SST) to verify precision and signal‐to‐noise ratio.

For sample preparation, an initial methanolic suspension of each drug substance batch is thoroughly mixed for 5 min and subsequently treated in an ultrasonic bath for 15 min. After the addition of water, the procedure is repeated. The resulting reaction mixture is then centrifuged for 5 min. The supernatant is filtered through a membrane filter, and the resulting filtrate is used for analysis.

Only 300 mg of the drug substance is required for sample preparation. However, just over 150 mL of methanol is needed in the sample preparation of the nitrosamine spike solution. The sample preparation itself involves numerous steps and is time‐consuming (at least 45 min per sample, based on the individual steps of Ph. Eur. Method A). None of the reagents or solvents used, with the exception of water, are considered of biological origin, but it should be noted that, for example, methanol could be produced from biogas. Nevertheless, as methanol is currently obtained from unsustainable sources, this was assessed with a negative impact on the AGREE score for all analytical methods that use methanol. The classification according to the AGREE system is illustrated in Figure 2 with individual values reported in Table 2.

**TABLE 2:** Individual values from AGREE for all compared methods; for variables that are not clearly defined, for example, when several possibilities could apply, the worst‐case scenario was always assumed.

Method	Ph. Eur. Method A	Ph. Eur. Method B Preparation 1	Ph. Eur. Method B Preparation 2	Ph. Eur. Method C	USP Procedure 1	USP Procedure 2	USP Procedure 3	USP Procedure 4	Schmidtsdorff et al.
1 Direct analytical techniques should be applied to avoid sample treatment	0.48	0.48	0.48	0.48	0.48	0.48	0.48	0.48	0.48
2. Minimal sample size and minimal number of samples are goals	0.82	0.75	0.65	0.75	0.98	0.88	1.0	0.75	0.78
3. If possible, measurements should be performed in situ	0	0	0	0	0	0	0	0	0
4. Integration of analytical processes and operations saves energy and reduces the use of reagents	0.8	1.0	1.0	1.0	1.0	1.0	0.8	0.8	1.0
5. Automated and miniaturized methods should be selected	0.5	0.5	0.5	0.5	0.5	0.5	0.5	0.5	0.5
6. Derivatization should be avoided	1.0	1.0	1.0	1.0	1.0	1.0	1.0	1.0	1.0
7. Generation of a large volume of analytical waste should be avoided, and proper management of analytical waste should be provided	0	0	0.02	0	0.26	0	0.26	0.13	0.06
8. Multi‐analyte or multi‐parameter methods are preferred vs. methods using one analyte at a time	0.48	0.48	0.48	0.76	0.59	0.45	0.72	0.81	1.0
9. The use of energy should be minimized	0	0	0	0	0	0	0	0	0
10. Reagents obtained from renewable sources should be preferred	0.5	0.5	0	0.5	0.5	0	0.5	0	0.5
11. Toxic reagents should be eliminated or replaced	0.16	0.8	0.79	0.8	0.18	0.32	0.34	1	0.29
12. Operator's safety should be increased	0.6	0.6	0.8	0.6	0.6	0.8	0.6	1	0.2

*Note*: If no information on the volumes of the solutions was provided, a standard volume of 10 mL was assumed, and toxic waste was calculated by run.

Abbreviations: AGREE, Analytical GREEnness Metrics; Ph. Eur., European Pharmacopoeia; USP, US Pharmacopoeia.

Similar to the above‐described method, Ph. Eur. Method B (GC–MS) prescribes two different sample preparation procedures depending on the tested drug substance. The primary difference between each procedure lies in the extraction mixture used. In sample Preparation 1, the extraction mixture is an aqueous solution of sodium hydroxide, containing the internal standard *N*‐nitrosoethylmethylamine (NEMA). Sample Preparation 2, on the other hand, is using dichloromethane as extraction solvent, spiked with the same internal standard (total volume 100 mL).

In sample Preparation 1, the substance is suspended in an aqueous solution of sodium hydroxide and then vigorously shaken for 5 min. Subsequently, it is extracted with dichloromethane for at least 5 min. After centrifugation for 5 min, the organic phase is collected and used directly.

In contrast, sample Preparation 2 involves suspending the substance directly in dichloromethane, followed by shaking for at least 5 min and centrifugation. The clear supernatant is then collected and, if necessary, filtered through a membrane filter.

Sample Preparation 1 requires 500 mg of the drug substance, whereas sample Preparation 2 requires 1000 mg. Compared to Ph. Eur. Method A, these sample preparation procedures are significantly less complex and less time‐consuming. However, they involve a higher proportion of hazardous reagents, namely, sodium hydroxide and dichloromethane. The classifications according to the AGREE system for both sample pretreatment procedures are separately illustrated in Figure 2 with individual values reported in Table 2.

Method C of the Ph. Eur. (GC–MS/MS) uses a sample preparation procedure similar to sample Preparation 1 for Ph. Eur. Method B, leading to the classification according to the AGREE system as illustrated in Figure 2 with individual values reported in Table 2.

Procedure 1 (LC–HRMS) from USP monograph 〈1469〉 uses a relatively simple sample preparation method for limit testing. In this procedure suitable for limit test, 100 mg of drug substance are dissolved or suspended in methanol and then filtered through a syringe filter. The resulting solution is used for analysis. Standard (6.0 ng/mL) and sensitivity (1.0 ng/mL) samples are prepared by mixing CRS of NDMA, NMBA, NDEA, NEIPA, NDIPA, NMPA, and NDBA in methanol. The classifications according to the AGREE system are illustrated in Figure 2 with individual values reported in Table 2.

Procedure 2 (HS–GC–MS) from USP monograph 〈1469〉 prepares samples for headspace injection. Methanol is used as the solvent. In addition to the internal standard (NDMA‐d_6_), a standard stock solution and a sensitivity solution are prepared (both as dilutions from a nitrosamine reference stock solution, which is prepared in the first step of the procedure and mixed with the internal standard). All samples are prepared in a headspace vial by adding a defined volume of the standard, sensitivity solution, or in the case of test samples, 200 mg drug substance. Additionally, approximately 100 mg imidazole, internal standard, and acetonitrile are added to all vials. A blank sample is prepared in the same way but omitting the drug substance. Again, the classifications according to the AGREE system for both sample pretreatment procedures are separately illustrated in Figure 2 with individual values reported in Table 2.

Procedure 3 (LC–MS/MS) of the USP is a quantitative method and therefore requires the preparation of a calibration curve consisting of five different calibration levels. These are prepared by diluting the previously prepared nitrosamine standard stock solution (containing CRS of NDMA, NEiPA, NDiPA, NDBA, and NMBA), the NDEA standard stock solution, and the internal standard. All solutions are prepared in water. The test sample is prepared by extracting 80 mg of drug substance with 0.1% aqueous formic acid. After vortexing for 20 min and centrifugation for 10 min, the sample is filtered and the filtrate is used for analysis.

Procedure 4 (GC–MS/MS) of the USP is also a quantitative method and similarly requires a calibration curve consisting of five calibration levels. The difference from Procedure 3 is that the internal standard solution, which uses dichloromethane as the solvent, is used for dilution. This results in the production of a large volume of halogenated organic waste. For sample preparation, 500 mg of drug substance is suspended in internal standard solution and vortexed for 1 min. After centrifugation for 2.5 min, an aliquot of the lower dichloromethane phase is filtered and used as the sample. The classifications according to the AGREE system for both sample pretreatment procedures are separately illustrated in Figure 2 with individual values reported in Table 2.

Schmidtsdorff et al. (SFC–MS/MS) employ a notably simple sample preparation procedure. The substance is suspended in methanol and shaken for 15 min. An aliquot of this suspension is then centrifuged for 5 min, and 1 mL of the resulting particle‐free supernatant is used as the test sample. In parallel, samples spiked with the 16 *N*‐nitrosamine impurities (CRS) are prepared in the same manner for limit test evaluation. The classification according to the AGREE system is illustrated in Figure 2 with individual values reported in Table 2.

### 3.2 Chromatography and Detection

As previously described in the earlier sections, all eight methods employ mass spectrometry–based detection. Method B from Ph. Eur. and Procedure 2 from USP rely solely on single‐stage mass analysis, whereas Methods A and C from Ph. Eur., USP Procedures 3 and 4, and the approach by Schmidtsdorff et al. utilize MS/MS techniques. Procedure 1 from USP in contrast uses HRMS. Consequently, the methods differ only slightly in terms of detection with respect to sustainability.

Three different separation techniques are used across the methods: LC (Method A, Procedures 1 and 3), GC (Methods B and C, Procedures 2 and 4), and SFC (Schmidtsdorff et al.). Individual classifications according to the AGREE system are illustrated in Figure 2 with individual values reported in Table 2.

Method A of the Ph. Eur. (LC–MS/MS) employs a C18 phase in combination with a mobile phase consisting of 0.1% formic acid in water (Eluent A) and methanol (Eluent B). The multistep gradient runs from 20% to 95% Eluent B over a period of 35 min, with a flow rate of 0.5 mL/min. Method A is used to analyze six different impurities (see Table 1; adopted from the Ph. Eur.). Detection is realized by MS/MS with an atmospheric pressure chemical ionization (APCI) probe in positive mode.

In contrast, Method B of the Ph. Eur. (GC–MS) only tests for the three target analytes NDMA, NEMA, and NDEA by GC–MS. Separation is achieved using a cyanopropylphenylene(6)‐methyl(94)‐polysiloxane phase and a temperature program (40–280°C) over a duration of 17 min. Helium is used as the carrier gas at a flow rate of 1.0 mL/min. Single mass detection is applied with electron ionization (EI).

Method C of the Ph. Eur. (GC–MS/MS) is applied both for limit testing and for quantitation of NDMA, NDEA, NEiPA, NDiPA, NDPA, and NDBA. Compared to Method B, only the helium flow rate is slightly increased (1.3 mL/min), and the temperature program is more complex, incorporating multiple heating rates and hold times. These optimizations reduce the total runtime to 14.0 min.

Procedure 1 of the USP (LC–HRMS) utilizes a pentafluorophenyl propyl stationary phase (USP column class L43) column and a mobile phase consisting of 0.1% formic acid in water (Eluent A) and 0.1% formic acid in methanol (Eluent B). The multistep gradient runs from 10% to 55% Eluent B over a period of 17 min, with a flow rate of 0.6 mL/min. The method allows for the simultaneous detection and quantitation of seven nitrosamines in three different sartan drug substances by HRMS with an electrospray ionization (ESI) probe in positive mode.

Procedure 2 of the USP (HS–GC–MS) focuses on quantitation of four different nitrosamines in all relevant sartan drug substances. Separation is achieved using helium as mobile phase on a fused‐silica stationary phase coated with a polyethylene glycol layer with a molecular weight of about 15 000 (USP column class G16). The temperature program from 45°C to 240°C is performed over a total run time of 30 min on a headspace GC system with single mass detection and EI.

Procedure 3 of the USP is an LC‐APCI‐MS/MS‐based method similar to Procedure 1 but conducted on a C18 column (USP column class L1). It applies a more complex gradient, running from 3% to 95% Eluent B over 12 min. Procedure 3 is particularly intended for quantitation of six nitrosamines in all relevant sartan drug substances.

Procedure 4 of the USP, a GC‐EI‐MS/MS method utilizing helium as carrier gas on the same column type, as Procedure 2, is intended for the quantitation of six nitrosamines in only three different sartans. Instead of a headspace injection, it applies direct injection and a less complex temperature stepwise gradient from 40°C to 250°C in approximately 13 min.

Schmidtsdorff et al. use a separation method on the basis of SFC, in which carbon dioxide is brought to a supercritical state and used as Eluent A. In addition to CO_2_, methanol (+0.1% TFA) is used as modifier (Eluent B), and a solution of 0.35% formic acid in methanol as the makeup solvent to transfer the analytes and to support ionization in the MS. A column with highly stable porous graphitic carbon is used as the stationary phase, with unique selectivity through π–π interactions and polar retention mechanisms. This makes it especially effective for separating polar, structurally similar, or isomeric compounds that are challenging for traditional reversed‐phase columns. The total runtime of the method, including re‐equilibration, is 11.5 min, with all 16 nitrosamine impurities being separated within the first 4 min and detected by ESI‐MS/MS in positive mode. This method can be used universally for all classes of drug substances and drug products [12, 23].

The use of the SFC approach was chosen, as it is able to combine advantages of GC and LC. For example, highly polar nitrosamines, like NMBA, are not volatile enough to be analyzed by GC (Table 1) or sometimes elute during the dwell volume of the LC. In addition, SFC can offer a higher chromatographic performance than LC, which allows the separation of structurally similar compounds. This is necessary to differentiate between, for example, dimethylformamide (DMF) that is not carcinogen and NDMA or NDPA and NDiPA, which have different acceptable intake thresholds (26.5 vs. 1500 ng/day) [16]. Most of the additional eight nitrosamines covered by the SFC–MS/MS, which are small aliphatic, aromatic, or cyclic nitrosamines mentioned in the EMA and FDA guidelines, should also be analyzable by the Ph. Eur. and USP methods, but it should be noted that especially the highly polar compounds like 1‐methyl‐4‐*N*‐nitrosopiperazine (MeNP), *N*‐nitroso‐diethanolamine (NDELA), and *N*‐nitrosopiperazine (NPZ) could be a challenge for them.

## 4 Discussion

A comparison of the different sample preparation methods reveals that the extraction media used in the GC methods with liquid injection (Ph. Eur. Methods B and C, USP Procedure 4) are classified less green than those used in the HPLC–MS/MS (Ph. Eur. Method A, USP Procedures 1 and 3) and SFC–MS/MS methods. The switch from liquid injection to headspace injection in USP Procedure 2 demonstrates a significant improvement in greenness compared to the other GC methods in terms of sample preparation and has a major impact on the overall outcome. With this type of sample preparation, conventional halogenated extraction solvents can be omitted, as the sample is simply extracted with methanol and the analytes are injected from the gas phase after heating the sample. This approach eliminates the need for extensive sample preparation, as the target analytes are transferred into the gas phase prior to injection into the system. Several other published methods show the advantage of this type of injection [24, 25, 26]. Even complete solvent‐free sample preparation is possible in this way, as demonstrated by Lee et al. [27].

A drawback of the sample preparation in Method A from the Ph. Eur. (LC–MS/MS) is the large number of preparation steps and the associated time consumption. In contrast, Schmidtsdorff et al. keep the sample preparation as simple and rapid as possible while avoiding the use of chlorinated solvents. These considerations are also reflected in the evaluation of the methods, as illustrated in Figure 2. Particular emphasis is placed on the solvents and reagents used, as well as the complexity of the sample preparation procedure.

When considering the separation techniques, it becomes evident that the method employing a supercritical mobile phase offers the advantage of a significantly shorter runtime while achieving high peak resolution and inclusion of additional target analytes. Compared to the longest method in this comparison (Ph. Eur. Method A, 34 min by LC–MS/MS), the runtime was reduced by 67%. This reduction allows for a higher sample throughput in a shorter time and substantially decreases the consumption of eluents. This can also be demonstrated by comparing the consumption of liquid eluents in LC and SFC methods. Considering only the consumption of methanol (Eluent B), it becomes evident that USP Procedure 3 (LC–MS/MS) has the lowest consumption at just 3.58 mL per run, followed by the SFC method with 5.99 mL per run, USP Procedure 1 with 6.88 mL (LC–HRMS), and, by far the highest, Method A of the Ph. Eur. (LC–MS/MS) with 12.44 mL per run. However, it should also be taken into account that in SFC, CO_2_ does not contribute to the overall solvent consumption (including aqueous eluents in HPLC methods), as it simply evaporates. In contrast, the HPLC methods shown require more energy at the source to evaporate the eluents.

Additionally, it is worth noting that Schmidtsdorff et al. (SFC–MS/MS) addressed a critical issue in the determination of NDMA. As the determination of NDMA may be affected by the isotopic mass of DMF in MS and MS/MS, chromatographic separation of NDMA and DMF is essential when using unit‐mass MS detection [28]. Method A of the Ph. Eur. explicitly warns of potential interference from DMF, whereas Schmidtsdorff et al. demonstrate successful separation of NDMA from DMF.

Given that the CO_2_ used as eluent is sourced from the natural carbon cycle and most likely returned to the environment in approximately equal amounts, the use of CO_2_ in SFC can be considered highly sustainable. A further improvement in ecological sustainability might be achieved by omitting or replacing TFA as an additive in Eluent B. However, Schmidtsdorff et al. report that 0.1% TFA in Eluent B (equal to about 6.0 µL per samples run) was necessary to ensure good peak shape and effective elution of drug substances from the column. Nevertheless, no further experiments were carried out, for example, to use more environmentally friendly chemicals such as difluoroacetic acid.

In contrast to the methods discussed above, the GC‐based methods (Ph. Eur. Ethods B, C and USP Procedures 2 and 4) use only one eluent, helium. Helium is obtained through fractional distillation of natural gas and is therefore not considered a sustainable resource. Moreover, the price of helium has increased significantly in recent years, which, along with the high energy consumption of GC systems, negatively impacts the economic sustainability of these four methods. To increase the sustainability of these methods, it would be reasonable to investigate whether helium can be replaced with hydrogen as the carrier gas. Hydrogen can be produced green or turquoise through various pathways, which would be advantageous in terms of sustainability.

As shown in Figure 2 (Section 9 “Energy Consumption”), all methods under comparison exhibit a negative energy balance. This is primarily due to the use of mass spectrometry, which is highly energy‐intensive. Although alternatives to MS exist, their applicability is limited, as the analytical target profile requires both a very low limit of detection and substance identification on the basis of the mass‐to‐charge ratio. Alternative detection methods like chemiluminescence nitrogen detection (CLND), nitrogen‐phosphorous detection (NPD), or the thermal energy analyzer (TEA) coupled to HPLC or GC are reported in the review article of Parr and Joseph [13]. However, they are considered not useful due to their limitations in sensitivity.

In total, the optimization of *N*‐nitrosamine analysis, as described by Schmidtsdorff et al., demonstrates a clear improvement in greenness as evaluated by measures of the AGREE system. This method covers 16 different *N*‐nitrosamines; the published methods cover from USP and Ph. Eur. only three to seven. Therefore, the method by Schmidtsdorff et al. also exhibits the highest analytical performance and is the only one among the compared methods to achieve the maximum value of 1.0 in Section 8. However, it should be noted that the number of *N*‐nitrosamines covered by the Ph. Eur. and USP methods can be increased theoretically, provided full method validation is performed. Nonetheless, due to inherent limitations in analyzing highly polar and non‐volatile compounds, it remains uncertain whether either technique can match the chromatographic performance of SFC.

Of course, not all impurities validated in the method will occur in a sample at the same time. However, the advantage of this method can still be seen in the fact that only one method, and therefore only one system, is required for testing several drug substances.

Consequently, it can also be stated that this method offers an economic advantage, as more analytes can be analyzed in less time. Additionally, the SFC–MS/MS was developed not only for sartans or drug substances only but also for drug product analysis, as mandatory based on EMA and FDA guidelines. The only drawback of this method is the use of TFA, leading to a very low score in Section 12 (operator safty; 0.2), which also drastically reduces the overall score. However, as previously discussed, it is not always possible to optimize a method across all parameters of green analytical chemistry. However, despite their favorable sustainability profiles, USP Procedure 1 and USP Procedure 3 are restricted to the analysis of sartans. Furthermore, USP Procedure 3, which utilizes tandem mass spectrometry rather than HRMS, does not permit chromatographic separation of DMF from NDMA. Therefore, the applicability of these greener methods is limited.

Overall, it can be stated that methods for the determination of *N*‐nitrosamines still need to be improved with respect to sustainability. There are several points of intervention that may be considered for method adaptation. For example, the choice of reagents and solvents plays a significant role. The use of sustainable reagents and solvents, such as biomethanol, which directly impacts the greenness of the methods or the exchange of organic solvents by greener solvents, for example, ethanol or even better bioethanol, or isopropyl alcohol, instead of the used reagents (e.g., dichloromethane) may significantly increase sustainability. One example can be the replacement of methanol with azeotropic ethanol in chiral analysis using SFC as shown by Roy et al. [29]. However, the latter changes will require partial revalidation and consecutive method performance verification. In this context, the application of a comprehensive approach to analytical life cycle management, as promoted by ICH Q14, is becoming increasingly important [30]. Lifecycle management ensures that method changes, including those aimed to improve environmental impact, are systematically evaluated, implemented, and controlled. It enables the continuous improvement of the analytical methods by integrating risk‐based planning, scientific justification, and method robustness evaluation. In addition, sustainable method adaptation guided by lifecycle principles supports both regulatory compliance and environmental responsibility.

Furthermore, the users’ health must be taken into account, as requested by SDG goal 3 (good health and well‐being). Therefore, efforts should be made to select chemicals that are as user‐friendly as possible or to minimize the use of potentially hazardous reagents and their potential impact not only on the laboratory personnel but also on the environment.

Method efficiency also plays a crucial role. Schmidtsdorff et al. have already demonstrated with their method that reducing method runtime can help to conserve both energy and resources.

On the other hand, further economic aspects of sustainability must also be considered. Not every facility has access to an SFC system or the means to acquire one. Additionally, the training status of the staff members needs to be considered. Hybrid systems combining SFC and HPLC may therefore play a more prominent role in the future, helping to minimize acquisition and maintenance costs. Similarly, it needs to be considered whether MS/MS instrumentation is already accessible, which is currently only limited in pharmaceutical QC laboratories. Moreover, the implementation or optimization of a method is not only time‐consuming but also not always economically viable. Users must assess how frequently such a method is employed and to what extent its optimization or implementation is financially beneficial for the organization.

It quickly becomes apparent that sustainability not only involves minimizing the ecological footprint but also requires consideration of the economic dimension and methodological efficiency. From this perspective, sustainability can be seen as a triangle of competing priorities, as illustrated in Figure 1 (Supporting Information).

## Conflicts of Interest

The authors declare no conflicts of interest.

## Supporting information




**Supporting File**: elps70067‐sup‐0001‐SuppMat.docx.

## Data Availability

Data are excerpts from referenced method descriptions that are publicly available.
