# Supplementary material for: Sustainability in Analytical Chemistry Illustrated by Pharmaceutical Nitrosamine Testing
Source: Electrophoresis. 2025 Dec 24;47(2):175–84. doi: 10.1002/elps.70067 (PMC12905522; doi:10.1002/elps.70067)
Supplement: Supplementary file 1 — Supporting File: elps70067‐sup‐0001‐SuppMat.docx. [file ELPS-47--s001.docx]

Supplementary Information: Selected parameters for the individual methods

| Method | Ph. Eur. Method A | Ph. Eur. Method B Preparation 1 | Ph. Eur. Method B Preparation 2 | Ph. Eur. Method C | USP Procedure 1 | USP Procedure 2 | USP Procedure 3 | USP Procedure 4 | Schmidtsdorff *et al.* |
| --- | --- | --- | --- | --- | --- | --- | --- | --- | --- |
| 1 Direct analytical techniques should be applied to avoid sample treatment. | off-line | off-line | off-line | off-line | off-line | off-line | off-line | off-line | off-line |
| 2. Minimal sample size and minimal number of samples are goals. | 0.3 g | 0.5 g | 1 g | 0.5 g | 0.1 g | 0.2 g | 0.08 g | 0.5 g | 0.4 g |
| 3. If possible, measurements should be performed in situ. | off-line | off-line | off-line | off-line | off-line | off-line | off-line | off-line | off-line |
| 4. Integration of analytical processes and operations saves energy and reduces the use of reagents. | 4 | <3 | <3 | <3 | <3 | <3 | 4 | 4 | <3 |
| 5. Automated and miniaturized methods should be selected. | automatic  not miniaturized | automatic  not miniaturized | automatic  not miniaturized | automatic  not miniaturized | automatic  not miniaturized | automatic  not miniaturized | automatic  not miniaturized | automatic  not miniaturized | automatic  not miniaturized |
| 6. Derivatization should be avoided. | - | - | - | - | - | - | - | - | - |
| 7. Generation of a large volume of analytical waste should be avoided, and proper management of analytical waste should be provided. | 175 ml | 1060 ml | 150 ml | 1060 ml | 25 ml | 160 ml | 25 ml | 70 ml | 110 ml |
| 8. Multi-analyte or multi-parameter methods are preferred versus methods using one analyte at a time. | 6 analytes  1.5 runs/h | 3 analytes  3 runs/h | 3 analytes  3 runs/h | 7 analytes  4 runs/h | 7 analytes  2 runs/h | 4 analytes  2 runs/h | 6 analytes  4 runs/h | 7 analytes  5 runs/h | 16 analytes  5 runs/h |
| 9. The use of energy should be minimized. | LC-MS | GC-MS | GC-MS | GC-MS | LC-MS | GC-MS | LC-MS | GC-MS | LC-MS |
| 10. Reagents obtained from renewable sources should be preferred. | some | some | none | some | some | none | some | none | some |
| 11. Toxic reagents should be eliminated or replaced | yes  14 ml | yes  0.0015 ml | yes  0.11 ml | yes  0.0025 ml | yes  12.5 ml | yes  4 ml | yes  3.5 ml | no | yes  5.34 ml |
| 12. Operator's safety should be increased. | highly flammable,  corrosive | highly flammable,  corrosive | highly flammable | highly flammable,  corrosive | highly flammable,  corrosive | highly flammable | highly flammable,  corrosive | - | highly flammable, toxic to aquactic life, persistent, corrosive |
